# Effect-Directed Profiling of Strawberry Varieties and Breeding Materials via Planar Chromatography and Chemometrics

**DOI:** 10.3390/molecules27186062

**Published:** 2022-09-16

**Authors:** Petar Ristivojević, Nevena Lekić, Ilija Cvijetić, Đurđa Krstić, Filip Andrić, Dušanka Milojković-Opsenica, Gertrud E. Morlock

**Affiliations:** 1 Chair of Analytical Chemistry & Center of Excellence for Molecular Food Sciences, Faculty of Chemistry, University of Belgrade, Studentski trg 12-16, 11158 Belgrade, Serbia; 2 Faculty of Agriculture, University of Belgrade, Nemanjina 6, Zemun, 11080 Belgrade, Serbia; 3 Chair of Food Science, Institute of Nutritional Science, and Interdisciplinary Research Center, Justus Liebig University Giessen, Heinrich-Buff-Ring 26-32, 35392 Giessen, Germany

**Keywords:** strawberry genotypes, HPTLC fingerprint, effect-direct analysis, principal component analysis, antioxidative activity, density functional theory calculation

## Abstract

Strawberries are an important fruit in the European diet because of their unique taste and high content of essential nutrients and bioactive compounds. The anthocyanins are known to be colorful phenolics in strawberries. In 17 samples of six strawberry cultivars produced in Serbia, i.e., the common varieties Alba, Asia, and Clery as well as promising breeding materials (11.29.11, 11.34.6, and 11.39.3), the anthocyanin profile as well as antimicrobial and antioxidative activity profiles were determined. All investigated extracts showed antioxidative and antibacterial activities against Gram-negative *Aliivibrio fischeri.* The responses were quite similar in number and intensity. The HPTLC-DPPH**•** scavenging assay and HPTLC-*Aliivibrio fischeri* bioassay coupled with high-resolution mass spectrometry identified pelargonidin-3-*O*-glucoside (Pg-3-glc) as the main anthocyanin and prominent antioxidative and antimicrobial compound in strawberries. The density functional theory calculations at the M06-2X/6-31+G(d,p) level showed that Pg-3-glc quenches free radicals via sequential proton loss electron transfer mechanism in water and in pentyl ethanoate, where the 5-OH group is the most reactive site for proton and hydrogen atom transfer. The results were confirmed via spectrophotometry. The highest total phenolic content was found in Clery and 11.39.3, while statistically significant differences between the genotypes regarding the antioxidant activity were not confirmed. Although very similar in the anthocyanin, antioxidative, and antimicrobial profile patterns, the strawberry genotypes were successfully classified using principal component analysis.

## 1. Introduction

Due to its attractive color, flavor, and aroma, strawberry fruits (*Fragaria x ananassa* Duch.) are frequently consumed in the human diet [1,2]. Suitable agroecological conditions in the various parts of Serbia provide possibilities for strawberry production (30,000–35,000 t) within the period from April in plain regions until August in hilly-mountainous areas. Strawberries are commercially produced on about 2700 ha with average yields of 3.7–5.5 t/ha, whereby part of the high-value production serves for the premium market and export [3,4]. Several factors such as genetics, environmental availability of elements, cultivation type, ripeness state, and storage conditions are known to affect the quality of strawberries, which are a rich source of antioxidants including flavonoids, phenolic acids, and anthocyanins. A high amount of ellagic acid, ellagitannins, gallotannins, proanthocyanidins, quercetin, and catechin were found in strawberries. In addition, they are rich in vitamins C, E, folic acid, β-carotene, melatonin, and saccharides. Benefits associated with polyphenols’ consumption are high antioxidant activity that protects cells and reduces the risk of cardiovascular diseases and cancer, improvement of glucoregulation, antimutagenic activity, and reduction in inflammation [2,5,6,7]. Analyzing the antioxidant activity in 12 different fruits, strawberries showed the highest antioxidant activity compared to other fruits, while vitamin C was found as the main antioxidant in strawberries [6]. In addition, strawberry juice and products exhibited high antioxidant capacity against superoxide radicals, hydrogen peroxide, hydroxyl radicals, and singlet oxygen free radicals.

The antioxidants quench reactive free radicals by one of the following mechanisms. For hydrogen atom transfer (HAT), the antioxidant (MOH) transfers the hydrogen atom directly to a radical (R·), forming a more stable MO· radical and neutral R-H species. The thermodynamic feasibility of the HAT mechanisms can be estimated from bond dissociation enthalpies (BDE):

BDE = *H* (MO·) + *H* (H·) − *H* (MOH)

Single electron transfer followed by proton transfer (SET-PT) is a two-step mechanism, where MOH first donates one electron to R· to form R^–^ anion and MOH^+^· radical cation. In the second step, the radical cation transfers a proton to R^–^ yielding R-H and MO· radical (the same products as for HAT). These processes are characterized by ionization potential (IP) and proton dissociation enthalpy (PDE):

IP = *H* (MOH^+^·) + *H* (e^–^) − *H* (MOH)

PDE = *H* (MO·) + *H* (H^+^) − *H* (MOH^+^·)

The third mechanism is sequential proton loss electron transfer (SPLET), in which the antioxidant dissociates to anion and proton, followed by electron transfer from anion to free radical. The first step of SPLET is quantified by proton affinity (PA), and the second step is related to electron transfer enthalpy (ETE):

PA = *H* (MO^–^) + *H* (H^+^) − *H* (MOH)

ETE = *H* (MO·) + *H* (e^–^) − *H* (MO^–^)

Hence, one aim of our research was to identify the most potent antioxidant and antimicrobial compounds in the strawberry extracts, and to determine the most probable mechanism of its antioxidative activity by studying the thermodynamics of HAT, SET and SPLET using DFT calculations. Effect-directed analysis (EDA) using high-performance thin-layer chromatography (HPTLC) combined with high-resolution mass spectrometry (HRMS) and chemometrics allows the characterization of bioactive compounds separated on the plate as well as the classification of samples based on their chemical composition and biological activity. HPTLC was recognized as a simple, high-throughput, and low-cost technique for the profiling of natural products and complex food mixtures. HPTLC provides several advantages compared to other liquid chromatographic techniques such as minimal/no sample preparation, simple detection, and a wide range of stationary and mobile phases, as well as in situ identification of bioactives [8].

This method could provide better insight into potential differences between samples, compared to classical in vitro assays. Since this approach has not been applied to strawberry profiling so far, 17 samples of six different strawberry cultivars were analyzed. Three standard varieties (Alba, Asia, and Clery) are commonly grown in Serbia, but there are also promising new breeding varieties with the main purpose of selection for premium quality, taste, aroma, and high antioxidative capacity. Examples of the latter are the three genotypes 11.29.11, 11.34.6, and 11.39.3, which are currently in the process of patent registration at the International Union for the Protection of New Varieties of Plants, and are expected to become good substitutes for current standard varieties. For quality measure and branding, it is important to recognize any bioactivity differences between the six different strawberry cultivars regarding the anthocyanin profile as well as antimicrobial and antioxidative activity profiles.

## 2. Results and Discussion

### 2.1. HPTLC Profiling

**Figure 1:**
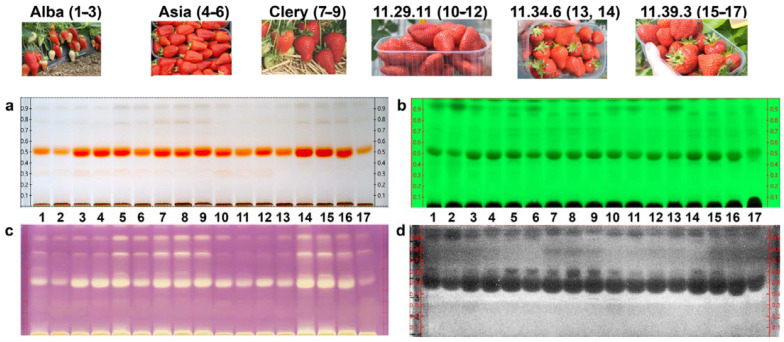
HPTLC profiles of 17 strawberry samples of six different cultivars (**a**) at white light illumination, (**b**) at 254 nm, (**c**) after the DPPH• scavenging assay and (**d**) *Aliivibrio fischeri* bioassay.

HPTLC profiling of 17 strawberry samples was performed to identify major anthocyanins and to find similarities/differences between the six different genotypes, i.e., Alba, Asia, Clery, 11.29.11, 11.34.6, and 11.39.3. The HPTLC profiling of the anthocyanins at white light illumination (Figure 1a) showed only slight differences in intensity and chemical pattern. All strawberry cultivars contained one high-intensity band at *hR*_F_ 50. This dominant anthocyanin band was preliminarily assigned to a pelargonidin (Pg) glycoside due to its red color hue. Cultivars such as Asia, Clery, and 11.39.3 showed the highest response and thus were considered as interesting cultivars. Further low-intensity bands at *hR*_F_ 80 and 90 were especially in Asia, Clery, 11.34.6 and 11.39.3 extracts (Figure 1a, no. 5–9, 14–16). A hardly visible red band at *hR*_F_ 30 was found in all strawberry extracts, except for the cultivars 11.34.6 and 11.39.3.

The HPTLC chromatogram at FLD 254 nm (Figure 1b) showed comparatively more differences in the phytochemical pattern for the six strawberry genotypes: the same, already mentioned prominent red-colored and now UV-absorbing band at *hR*_F_ 50 was detected in all samples. Alba, Asia, 11.29.11 and one sample of 11.34.6 had another UV-absorbing band at *hR*_F_ 95. In some genotypes, such as Asia, Alba, and one sample each of 11.34.6 (no. 14) and 11.39.3 (no. 15), another very weak band at *hR*_F_ 44 was observed, while Asia, Clery, and 11.39.3 contained additionally one at *hR*_F_ 85. Our results were in accordance with the literature, in which Pg was identified as main anthocyanidin in strawberries by TLC, while cyanidin (Cy) was found in a smaller quantity [9], or in which Cy-3-glc, Pg-3-glc, and Pg-3-rut were found as predominant anthocyanins in strawberries by high-performance liquid chromatography [10,11,12,13]. In addition, Kelebek and Selli investigated the chemical profile, phenolic content and antioxidant activity of Camarosa, Seyhun, and Osmanli cultivars in Turkey. Pelargonidin-3-glucoside was the most dominant anthocyanin in all three cultivars [14]. Furthermore, a comprehensive and fully validated high-performance liquid chromatography tandem mass spectrometry method was developed for the simultaneous determination of 36 phenolic compounds such as anthocyanins, flavonols, flavones, flavan-3-ols, proanthocyanidins and phenolic acids in strawberry and blueberry fruits and their commercial jams, and to evaluate their antioxidant activities. The most abundant anthocyanins in blueberry and strawberry were delphinidin-3-*O*-galactoside (381 mg/kg) and pelargonidin-3-glucoside (180 mg/kg), respectively [15].

### 2.2. Effect-Directed Analysis

For the non-target effect-directed profiling, five different bioassays, i.e., the AChE and BChE inhibition assays, the Gram-positive *Bacillus subtilis* and Gram-negative *Aliivibrio fischeri* bioassays as well as the DPPH**•** scavenging assay, were applied to detect any bioactive compounds in the 17 strawberry extracts. The HPTLC-DPPH• assay and the *A. fischeri* bioassay showed bioactivity patterns that were highly comparable between the samples (Figure 1c–d). The DPPH• assay showed up to four individual bands as a yellow zone against a purple background (Figure 1c). All tested samples showed a similar pattern with the strongest radical scavenging zone at *hR*_F_ 50 and further with similar intensity (Figure 1c). The cultivars Asia, Clery, 11.39.3 and 11.34.6 (no. 14) showed a comparatively higher antioxidative activity and few weaker bands at higher *hR*_F_ values between 80 and 92.

Three zones active against Gram-negative bacteria were detected as dark zones against a grey background in the HPTLC–*A. fischeri* bioautogram (Figure 1d). All investigated strawberry extracts showed antibacterial activity and had a quite similar compound pattern regarding the number and intensity of the active compounds. The most pronounced antibacterial zone at *hR*_F_ 50 (broader zone due to diffusion) was instantly detectable, indicating a strong acute effect. Several strawberry cultivars such as Alba, one Asia sample, Clery, and 11.39.3 showed two further weaker antibiotic zones at *hR*_F_ 60 and 90.

The *Bacillus subtilis* bioautogram as well as the AChE and BChE inhibition bioautograms did not show a clear bioactivity, also not for the zone at *hR*_F_ 50, at the same amount applied (Figure S1, Supplementary material). Of course, the chromatographic system and sample amount can be further optimized to better detect these effects.

### 2.3. Characterization of the Main Bioactive Compound by HPTLC–UV/vis-HESI–HRMS/MS

**Figure 2:**
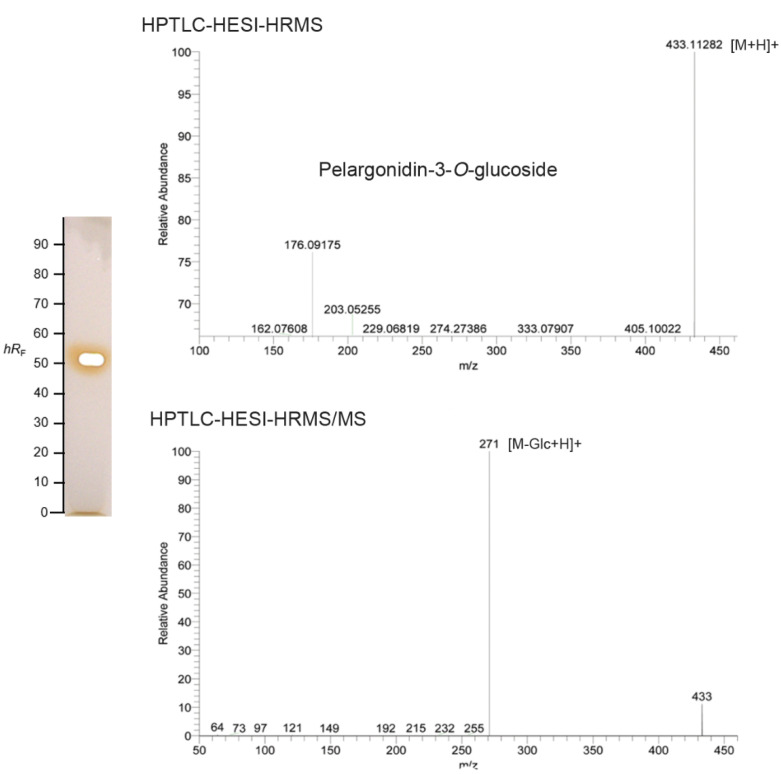
HPTLC-HESI-HRMS and fragmentation spectra of the red, UV-active, antibacterial and antioxidative zone at *hR*_F_ 50 (image after zone elution), assigned as pelargonidin-3-*O*-glucoside.

The main red band at *hR*_F_ 50 showed a characteristic UV/Vis spectrum with a maximal wavelength at about 500 nm, which was lower compared with other anthocyanins. Densitometric measurement of the HPTLC chromatograms confirmed it as the main compound (Figure S2). This red compound zone at *hR*_F_ 50 was also the most potent antioxidative and antimicrobial compound present in the strawberry cultivar extracts. Sample no. 15 was selected as a representative sample for online elution and subsequent characterization. The respective high-resolution mass spectra recorded within a minute showed a molecular ion at *m/z* 433.1128 (∆ppm 0.23), which was assigned to the protonated molecule [M+H]^+^ of pelargonidin-3-*O*-glucoside (Pg-3-glc, theoretical mass *m/z* 433.1129) and thus identified as the main strawberry anthocyanin (Table S1). The characteristic fragment ion at *m/z* 271 was obtained by loss of the glucose unit (Figure 2) [1,13]. The most prominent peak in the HPTLC chromatograms in all 17 samples corresponded to Pg-3-glc, which was in accordance to other studies, where Pg-3-glc was identified as the main anthocyanin in strawberries from Chile and Spain [1,16] or reported to be highest in the maturity stage of strawberries, while ellagic acid was found as major phenol in the green fruit [16,17].

### 2.4. Total Phenolic Content and Radical Scavenging Activity

**Figure 3:**
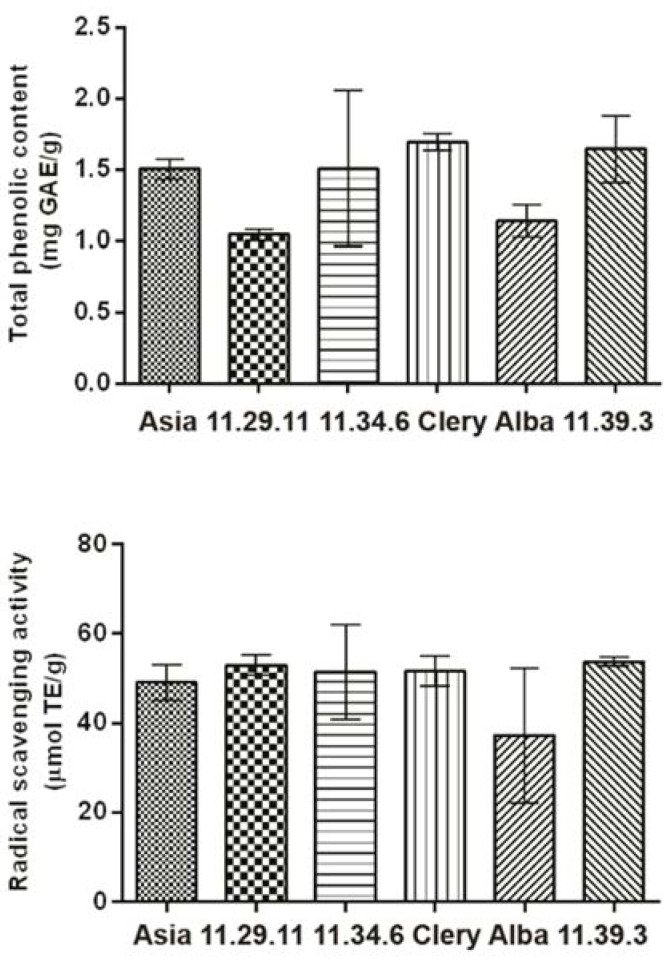
Mean total phenolic content and radical scavenging activity of six sorts of strawberry cultivars.

The total phenolic content (TPC) via the Folin–Ciocalteu method and the radical scavenging activity (RSA) of the 17 strawberry extract samples were determined. There was no significant difference in the RSA results between the six strawberry cultivars. The average RSA values of Asia (49.08 ± 3.9 μmol TE/g), 11.29.11 (53.0 ±2.4 μmol TE/g), 11.34.6 (51.5 ± 10.7 μmol TE/g), Clery (51.7 ± 3.4 μmol TE/g), Alba (37.3 ± 15.1 μmol TE/g) and 11.39.3 (53.7 ± 1.1 μmol TE/g) were similar. This agrees with the HPTLC-DPPH**•** assay, where a similar amount of Pg-3-glc, identified as the main radical scavenger, was found in all 17 strawberry samples. According to the TPC results, Clery (1.69 ± 0.06 mg GAE/g) and 11.39.3 (1.64 ± 0.24 mg GAE/g) had the highest content of these phytochemicals, followed by 11.34.6 (1.51 ± 0.55 mg GAE/g), Asia (1.50 ± 0.07 mg GAE/g) and Alba (1.14 ± 0.11 mg GAE/g). The lowest TPC value was measured for the 11.29.11 samples (1.04 ± 0.03 mg GAE/g). These results (Figure 3) were in agreement with another study, where there was no difference in the antioxidative activity of Alba (93.96 ± 0.69%), Clery (94.37 ± 0.70%), and Asia (94.95 ± 0.39%) [18].

### 2.5. Mechanism of Antioxidant Activity of Pg-3-glc Studied by DFT

**Figure 4:**
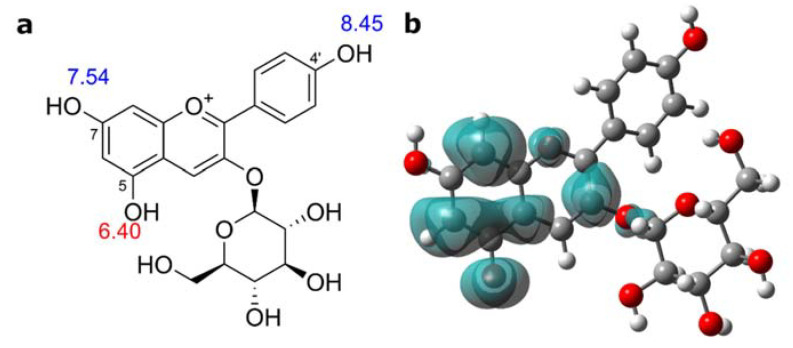
(**a**) Predicted p*K*_a_ values and (**b**) spin density distribution for 5–*O* radical of Pg-3-glc in water.

Since Pg-3-glc was identified as the major contributing compound to the overall antioxidative activity of strawberry extracts, it was of interest to study its mechanism. Although Pg-3-glc has three phenolic OH groups ionizable at physiological pH, its fully protonated forms were studied first since it is the most likely protonation state in less polar environments such as the interior of biological membranes. The BDE, IP, and PA values represent the first step of HAT, SET-PT, and SPLET mechanisms, respectively. The results (Table 1) show that the thermodynamically preferable antioxidant mechanism for a molecular form of Pg-3-glc is HAT in the gas phase, and SPLET in water and pentyl ethanoate. The p*K*_a_ predictions indicate that Pg-3-glc exists predominantly as monoanion in an aqueous solution at pH 7.4 (Figure 4a). The most favorable proton dissociation site is the 5-OH group. Proton affinities calculated at the DFT level corroborate this result (Table 1). According to BDEs, this group is also the most reactive hydrogen atom donor site of Pg-3-glc, ascribed to the high delocalization of the spin density of the 5–*O* radical (Figure 4b). Preliminary calculations for the Pg-3-glc monoanion suggest that both HAT and SET-PT mechanisms are facilitated compared with its molecular form. The BDEs for 7-OH and 4′-OH group of Pg-3-glc monoanion are 20–30 kJ/mol lower compared with molecular form while IPs are decreased even more dramatically, by 69 kJ/mol in the water and by 314 kJ/mol in the gas phase (Table 1 and Table S1). Therefore, ionization of Pg-3-glc at a physiological pH assumedly improves its ability to transfer a hydrogen atom or electron to reactive free radicals.

### 2.6. Principal Component Analysis

Principal component analysis (PCA) is a widely used multivariate analytical statistical technique that can be applied to multivariate data to reduce the set of original variables (*i.e*., phenolic compounds) to a much smaller set of latent, orthogonal variables (*i.e*., principal components, PCs) based on patterns of correlation among the original ones [19]. In the present case, PCA was applied to the data matrix (17 samples × 217 variables) obtained from the HPTLC plate, as an initial exploratory technique. Mean centering, warping and standard normal variate were applied as signal preprocessing techniques.

**Figure 5:**
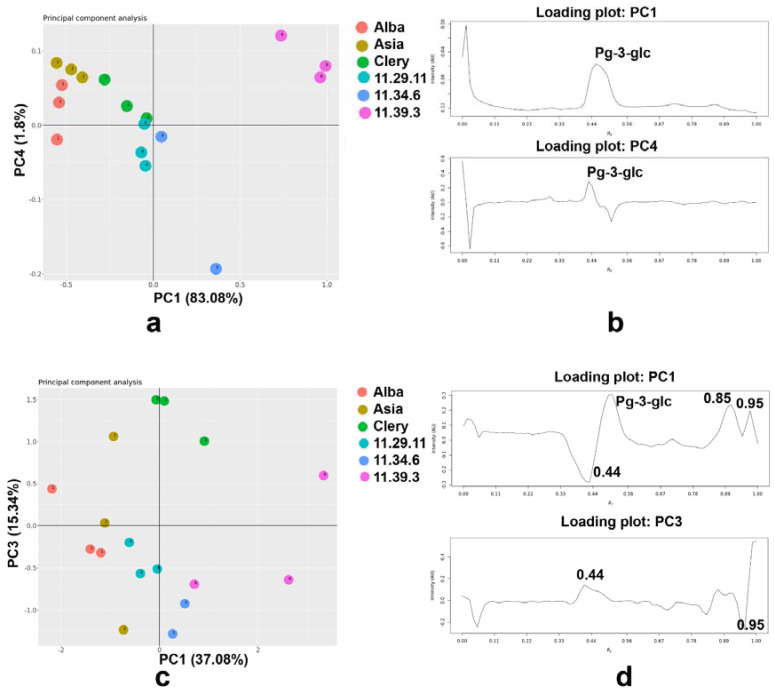
PCA based on the HPTLC fingerprint under white light illumination with (**a**) PCs score and (**b**) loading plot as well as under 254 nm with (**c**) PCs score and (**d**) loading plot.

The projections of the sample scores along the first and fourth principal components (PCs) were illustrated for the HPTLC images obtained under visible light, filtered through the grayscale (Figure 5a). The three varieties and three promising breeding materials of strawberries were separated, and there was also a clear separation between Alba, Asia, and 11.39.1. The promising breeding material 11.39.1 was separated from the others, while Clery, 11.29.11, and 11.34.6 did overlap (Figure 5a). The percent of the cumulative contribution of the variance of the first and fourth PCs was 84.88%, which was high enough to represent all the variables, while PC1 and PC4 expressed 83.08% and 1.8% of the total variability, respectively, to which Pg-3-glc contributed highly (Figure 5b). PC2 accounted 7.83%, while PC3 accounted 4.95% of the total variability. PCs score plots based on white light illumination (PC1 vs. PC2 and PC1 vs. PC3) and 254 nm (PC1 vs. PC2 and PC1 vs. PC4) were presented in Figure S3.

In the case of the HPTLC images inspected at 254 nm, and chromatographic signals acquired after grayscale filtering, warping, SNV, and mean centering, the three-component model was obtained. The PC 1 and PC2 described 37.08% and 33.08% of the variability, respectively, while the first three PCs described 85.50% of the total variability. By examining the PC score domain, good separation between the six different strawberry genotypes was observed, with some overlap of Asia with Alba and 11.29.11 samples (Figure 5c). By examining the loading plot (Figure 5d), PC1 was positively contributed by compounds with *hR*_F_ values at 44, 85, 95, and Pg-3-glc, while PC3 was highly dominated by compounds with *hR*_F_ values at 44 and 95 (Figure 5d).

**Figure 6:**
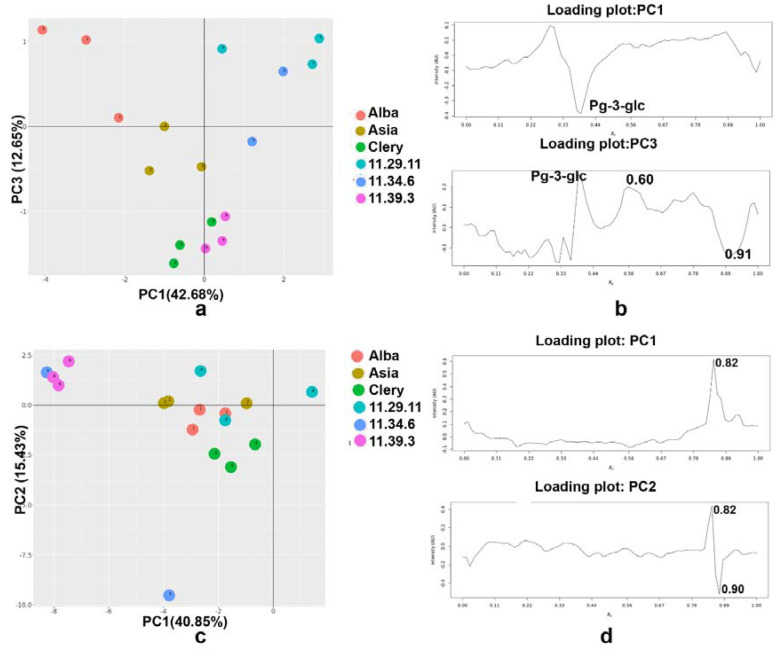
PCA based on effect-direct analysis via *Aliivibrio fischeri* bioassay with (**a**) PCs score and (**b**) loading plot as well as via HPTLC-DPPH**•** assay with (**c**) PCs score and (**d**) loading plot.

The PCA of the HPTLC–*A. fischeri* bioautograms (after grayscale HPTLC image filtering, baseline correction, COW, SNV, and autoscaling) resulted in a four-component model that explained 86.23% of the total data variance. PC1 described 42.68% of the total variability while PC2, PC3, and PC4 accounted for 25.88%, 12.65%, and 5.02%, respectively. The PC scores of strawberries extracts were projected along with the three PC directions, *i.e*., for the 2D representation, the PC1 vs. PC3 was selected (Figure 6a). The separation between promising breeding materials and three usual sorts of strawberries was evident (Figure 6a). However, the two 11.34.6 extracts overlapped with the 11.29.11 extracts. There was no clear separation between the usual strawberry cultivars because of the similar bioautographic pattern (Figure 6a). Inspection of the loading plot points to the Pg-3-glc, and compounds with *hR*_F_ values at 60 and 91 as a characteristic marker with potential Gram-positive antimicrobial activity (Figure 6b). PC2 accounted 7.83%, while PC3 accounted 4.95% of the total variability. PCs score plots based on white light illumination (PC1 vs. PC2 and PC1 vs. PC3) and 254 nm (PC1 vs. PC2 and PC1 vs. PC4) were presented in Figure S3.

Regarding the PCA of the HPTLC-DPPH**•** autograms, PC1 accounted for 40.85%, and PC2 for 15.43%, PC3 for 12.53%, PC4 for 6.70% of the total variance (Figure 6c). In this case, two signal preprocessing techniques such as COW and mean centering were enough to improve the multivariate model. The two promising varieties, 11.34.6 and 11.39.3 were separated from other samples, while 11.29.11, Asia, Alba, and Clery varieties formed one cluster (Figure 6c), which agrees with the similar HPTLC-DPPH**•** profiles. PC1 and PC2 were highly influenced by compounds at *hR*_F_ 82 and 90 (Figure 6d). PCs score plots based on *A. fischeri* (PC1 vs. PC2 and PC1 vs. PC4) and DPPH**•** scavenging (PC1 vs. PC3 and PC1 vs. PC4) were presented in Figure S3.

## 3. Material and Methods

### 3.1. Reagents and Chemicals

Fast Blue B salt, 2,2- diphenyl-1-picrylhydrazyl radical (DPPH, 97%), butyrylcholinesterase (BChE, from equine serum, ≥140 U/mg) and acetylcholinesterase (AChE, from *Electrophorus electricus* Linnæus, ≥245 U/mg), 1-naphthyl acetate, gallic acid, Trolox and solvents/reagents of analytical grade were purchased from Fluka Sigma-Aldrich, Schnelldorf, Germany. Bioluminescent marine *Aliivibrio fischeri* bacteria (NRRL-B11177, DSM no. 5171) were obtained from the German Collection of Microorganisms and Cell Cultures, Berlin, Germany. Ethanol, methanol and MS grade solvents (Optima LC-MS grade) methanol and acetonitrile were provided from Thermo Fisher Scientific, Schwerte, Germany. *Bacillus subtilis* spores (BGA, DSM 618 strain), formic acid (96%), hydrochloric acid, ethyl acetate, butanone, Folin-Ciocalteu reagent and HPTLC plates silica gel 60 F_254_ were obtained from Merck, Darmstadt, Germany. Thiazol blue tetrazolium bromide (3-(4,5-dimethylthiazol-2-yl)-2,5-diphenyl-tetrazolium bromide, MTT) were purchased from Carl Roth, Karlsruhe, Germany. Bidistilled water was prepared with a Destamat Bi 18E, Heraeus, Hanau, Germany. Formic acid (98%) was from J.T. Baker, Deventer, Netherlands.

### 3.2. Samples Preparation

Strawberries from selected cultivars (Alba, Asia, and Clery) and promising breeding materials (11.29.11, 11.34.6, and 11.39.3) were grown at the experimental station of Zeleni hit company, Belgrade, Serbia, which is a part of the BerryLab breeding consortium (Table 2). Samples were collected in the year 2019, whereas fruits were picked at the stage of commercial maturity. After harvest, fruits were washed in water, frozen, and stored at -20 °C until analysis.

### 3.3. Extraction of Phenolic Compounds

Phenolic compounds were extracted from 5 g frozen strawberry samples with a methanol−water mixture (70:30, *v/v*) acidified with 0.1% hydrochloric acid. Extraction was performed in an ultrasonic bath (Sonic, Niš, Serbia) for 1 h and centrifuged at 8000 rpm (Centrifuge, SL 16, Thermo Scientific Fischer, Dreieich, Germany) for 15 min. Two re-extractions were carried out. Pooled supernatants were evaporated to dryness (RV 05 basic Rotary Evaporator, IKA, Staufen, Germany) under reduced pressure at 35 °C. Obtained residues were dissolved in 10 mL methanol and stored at 4 °C. The samples were filtered through 0.45-μm membrane syringe filters (PTFE, Supelco, Bellefonte, PA, USA) before further analysis [20].

### 3.4. Determination of Total Phenolic Content (TPC)

The TPC was determined via the Folin–Ciocalteu method [21,22,23]. The spectrophotometric measurements were performed on a GBC UV-Visible Cintra 6 spectrophotometer (Dandenong, VIC, Australia). All the extracts were diluted to fit the calibration range and gallic acid was used as standard in the range of 20–100 mg/L. The volume of 0.5 mL of each extract and 0.5 mL of ultrapure water were mixed with 2.5 mL of 10% Folin-Ciocalteu reagent and incubated for 5 min. Subsequently, 7.5% sodium carbonate (2.0 mL) was added. The absorbance was measured at 765 nm after incubation of the mixture for 2 h at room temperature. TPC values were expressed as mg gallic acid equivalent (GAE) per kg frozen sample. All measurements were done in duplicate and the results were expressed as mean values ± standard deviation (SD).

### 3.5. Determination of the Radical-Scavenging Activity (RSA)

Radical scavenging activity was determined using the DPPH**•** solution. A 0.1 mL of the extracts was mixed with 4 mL of methanol solution of DPPH**•** (71 μM). The mixture was incubated for 1 h at room temperature and absorbance was measured at 517 nm. Trolox was used as standard in the range of 100–600 μmol/L. The obtained calibration curve was displayed as a function of the percentage of DPPH**•** scavenging [21,22,23]. The results were expressed as micromoles of Trolox equivalents per gram of frozen strawberries (µmol TE/g FW). The results were presented as mean values of two measurements ± standard deviation (SD).

### 3.6. High-Performance Thin-Layer Chromatography

The plate was prewashed with methanol and heated at 120 °C for 20 min. The methanolic extracts (15 µL) were applied as an 8-mm band (dosage speed 150 nL/s, Automatic TLC Sampler 4, CAMAG, Muttenz Switzerland). On the HPTLC plate silica gel 60 F_254_ (20×10 cm, no. 105641, Merck) 17 tracks were applied, whereby the distance between bands was 10.6 mm, first position Y was 8 mm, and X position was 13 mm. The Automatic Developing Chamber 2 was used for standardization of the plate activity with a saturated potassium acetate solution for 6 min that reached 43% humidity of the air passing the plate. A mixture of ethyl acetate–butanone–water–formic acid 7:3:0.8:1.2, *v/v/v/v*, was used as the mobile phase. After separation, the HPTLC plate was dried for 3 min [24].

### 3.7. Effect-Directed Analysis

For effect-directed analysis, the HPTLC chromatogram was dried for 15 min under cold air and/or phosphate buffer pH 7.8 (3 mL, speed 6) was used to neutralize acidic traces from the mobile phase development of the HPTLC chromatogram. For the DPPH• assay [25], the HPTLC chromatogram was dipped into a 0.05% methanolic DPPH• solution, dried (60 °C, 1 min), and recorded under white light illumination in the reflectance mode (TLC Visualizer, CAMAG). The images were captured again on the next day, as the signal intensity increased over time. For the *A. fischeri* bioassay [26], the HPTLC chromatogram was immersed into the bioluminescent Gram-negative *A. fischeri* bacteria suspension (TLC Immersion Device, CAMAG). The bioluminescence was depicted as a greyscale image (BioLuminizer, CAMAG). According to the cholinesterase inhibition assay method [27], the plate was sprayed with 1 mL Tris-HCl buffer (pre-wetting), sprayed (green nozzle, level 6, Derivatizer, CAMAG) with 3 mL AChE or BChE enzyme solution (2.7 mg AChE or 2.4 mg BChE in 100 mL Tris-HCl buffer plus 100 mg BSA), incubated for 25 min, sprayed with 0.5 mL of a mixture of 1-naphthyl acetate (4.5 mg in 1.5 mL ethanol) and then Fast Blue B salt solution (9 mg in 3 mL water). The plate was dried for 3 min to detect colorless zones on a purple background under white light illumination. For the B. subtilis bioasssay [28], the HPTLC chromatogram was sprayed with phosphate buffer at pH 7.8 (3 mL, speed 6), dried and then B. subtilis suspension (3 mL, speed 6), incubated for 90 min and sprayed with an aqueous solution of MTT solution and incubated for 30 min.

### 3.8. Characterization of Active Zones via HPTLC-HESI-HRMS

The sample was applied threefold on an HPTLC plate, prewashed with methanol-formic acid (10:1, *v/v*) and acetonitrile-methanol (2:1, *v/v*), and dried at 120 °C for 30 min. After chromatography, anthocyanins were marked with a pencil and eluted via PlateExpress (Advion, Ithaca, NY, USA) to the HESI source of the Q Exactive Plus (Thermo Fisher Scientific). Full scan mass spectra were recorded in the positive mode based on parameters described [29].

### 3.9. Multivariate Analysis

Principal component analysis was performed with the open-source software rTLC (http://shinyapps.ernaehrung.uni-giessen.de/rtlc/, accessed on 15 June 2022) [27]. PCA was carried out as an exploratory data analysis by using a singular value decomposition algorithm (SVD) and a 0.95 confidence level for Q and T2 Hotelling limits for outliers. Hierarchical cluster analysis (HCA) was obtained using the Ward method to calculate cluster distances and by applying Euclidean distance as a measure of distance between the samples.

### 3.10. Molecular Modeling

The initial 3D structure of Ppg-3-glc is retrieved from PubChem. It was first optimized using the PM7 semiempirical quantum chemical method [30] implemented in MOPAC2016 [31]. The solvation effects were simulated using the COSMO model of water. The PRECISE keyword was used to increase the convergence criteria. VegaZZ 3.2.0 was used as a GUI [32]. The p*K*_a_ values of Pg-3-glc were predicted using ChemAxon’s Marvin software.

DFT calculations were performed using M062X, since it proved to be highly accurate for predicting thermodynamic aspects of the antioxidant activity for many compounds [33]. The 6-31+g(d,p) basis set was used, and solvation effects were simulated using the SMD models of water and pentyl ethanoate. The enthalpies for solvated e^–^ and H^+^ ion were taken from the literature [34]. Frequency calculations confirmed the absence of imaginary vibrational frequencies in optimized geometries and provided zero-point energy and thermal enthalpy corrections that are necessary for studying the thermodynamics of the antioxidant activity of Pg-3-glc. All calculations were done in Gaussian 16, version B.01 [35].

## 4. Conclusions

Compared to conventional liquid column chromatography, spectrophotometry, or microtiter plate assays, which provide only a sum parameter, the newly developed HPTLC-UV/Vis-EDA-chemometrics approach is useful and reliable for simple, fast, and low-cost quality assessment and quality control of strawberry cultivars as well as for authentication of commercial and new strawberry genotypes. A classification of the samples was achieved using the physico-chemical, antioxidative and antimicrobial HPTLC profiles. As the most important radical scavenging and antibacterial compound (against Gram-negative *A. fischeri* bacteria), Pg-3-glc was identified in the strawberry cultivars. This compound is a potent radical scavenger via the SPLET mechanism, and its activity increases upon ionization at physiological pH. Based on TPC and RSA, some genotypes such as Clery and 11.39.3 were marked as rich in total phenolic content, while all six genotypes showed similar antioxidative activity.

## Figures and Tables

**Table 1:** Bond dissociation enthalpy (BDE), ionization potential (IP), proton dissociation enthalpy (PDE), proton affinity (PA), and electron transfer enthalpy (ETE) calculated for the molecular form of pelargonidin-3-*O*-glucoside at M062X/6-31+g(d,p) level in the gas phase, and using SMD solvation models of water and pentyl ethanoate. All values are given in kJ/mol.

Site	Gas Phase					Water					Pentyl Ethanoate				
	BDE	IP	PDE	PA	ETE	BDE	IP	PDE	PA	ETE	BDE	IP	PDE	PA	ETE
4′-OH	409.3	988.1	734.0	1024.8	697.2	407.3	524.1	41.8	96.1	469.9	402.8	630.6	15.6	123.2	523.0
5-OH	383.2		702.2	1016.2	674.1	384.9		19.4	88.0	455.5	376.2		−10.9	114.7	505.0
7-OH	414.9		728.3	1041.5	675.0	400.0		34.5	116.4	442.3	396.1		8.9	140.5	499.1

**Table 2:** Main characteristics of six genotypes of investigated strawberries.

ID	Genotype Patent No. Material Type	Main Characteristics	Use
1	Alba NF 311 Variety	Variety with very early season of ripening, characterized by high productivity and yield. Fruits are large, very uniform, with intense red color and average taste. Soluble Solids Content (°Brix) is lower than in new, promising breeding materials, and usually without ideally balanced sugar/acidity ratio. Due to its good shelf-life and consistency, variety is well suitable for long-distant markets.	Fresh market
2	Asia NF 421 Variety	Variety with medium-late season of ripening, characterized by plants of erect and vigorous habitus. The flavor is good, with well balanced sugar/acidity ratio. Variety is sensitive to powdery mildew, but well resistant to frosts.	Fresh market
3	Clery - Variety	Variety with very early season of ripening and medium productivity. Fruits are firm, intense red in color and with long-conical fruits of standard good taste. Variety is sensitive to high temperatures and late spring frosts. However, it is characterized as resistant to most strawberry root diseases.	Fresh market
4	11.29.11 BL 29 Breeding material	Perspective new genotype with very early season of ripening and high productivity and yield. Fruits are large, long-conical in shape, with very attractive bright color and great consistency, which suggests them for long-distant markets. It is characterized by premium quality fruits of good taste and well-balanced sugar/acidity ratio. Due to its resistance to rain, it is adapted for both open field and greenhouse cultivation.	Fresh market, organic cultivation, gardening
5	11.34.6 BL 34 Breeding material	Perspective new genotype with extremely early season of ripening, especially in cases of cultivation under tunnels or in greenhouses. Genotype is characterized by strong and rustic plants, medium productivity and without susceptibility to common strawberry fungal diseases. Fruits are uniform, long-conical in shape, bright-red colored, very sweet and aromatic of premium quality. This genotype is suitable for organic cultivation and resistant to rain damage.	Fresh market, organic cultivation, gardening
6	11.39.3 BL 39 Breeding material	Perspective new genotype with early season of ripening, strong plants, and medium/high productivity. Plants are described as rustic and strong, as well as not susceptible to common strawberry diseases. Fruits are short conical-round in shape, firm, very uniform, intensively bright-red colored, with premium quality and great shelf life. Due to its great organoleptic characteristics and interesting aroma, this genotype is suitable for industry/processing. Due to its resistance to rain, it is adapted for both open field and greenhouse production, but also suggested for organic cultivation.	Fresh market, processing, organic cultivation, gardening

## Data Availability

The data presented in this study are available on request from the authors.

## Supplementary Materials

The following supporting information can be downloaded at: https://www.mdpi.com/article/10.3390/molecules27186062/s1. Table S1: antioxidant mechanism prediction calculated for pelargonidin-3-*O*-glucoside monoanion; Figure S1: HPTLC profiles after *Bacillus subtilis*, AChE and BChE inhibition assays; Figure S2: HPTLC densitogram of the absorbance measurement; Figure S3: PCs score plots based on chromatogram at white light illumination (a), absorbance chromatogram at 254 nm (b), DPPH• scavenging autogram (c), and *Aliivibrio fischeri* bioautogram (d).

## Sample Availability

Food samples are not available from the authors.
