# Supplementary material for: Effect-Directed Profiling of Strawberry Varieties and Breeding Materials via Planar Chromatography and Chemometrics
Source: Molecules. 2022 Sep 16;27(18):6062. doi: 10.3390/molecules27186062 (PMC9503288; doi:10.3390/molecules27186062)
Supplement: Supplementary file 1 [file molecules-27-06062-s001.zip › molecules-1837685-supplementary.pdf]

Supplementary information

Effect-directed profiling of strawberry varieties and breeding materials via planar chromatography and chemometrics

Petar Ristivojević [°1], Nevena Lekić[°2], Ilija Cvijetić[1], Đurđa Krstić [1], Filip Andrić [1], Dušanka Milojković-Opsenica [1] and Gertrud E. Morlock [3,*]

[1] Chair of Analytical Chemistry & Center of Excellence for Molecular Food Sciences, Faculty of Chemistry, University of Belgrade, Studentski trg 12-16, 11158 Belgrade, Serbia; ristivojevic@chem.bg.ac.rs (P.R.); ilija@chem.bg.ac.rs (I.C.); djurdjakrstic@chem.bg.ac.rs (Đ.K.); andric@chem.bg.ac.rs (F.A.); dusankam@chem.bg.ac.rs (D.M.-O.)

[2] Faculty of Agriculture, University of Belgrade, Nemanjina 6, Zemun, 11080 Belgrade, Serbia; nevena.momirovic@agrif.bg.ac.rs

[3] Chair of Food Science, Institute of Nutritional Science, and Interdisciplinary Research Center, Justus Liebig University Giessen, Heinrich-Buff-Ring 26-32, 35392 Giessen, Germany

* Correspondence: gertrud.morlock@uni-giessen.de

† Equal contribution.

Content

Table S1. Bond dissociation enthalpy (BDE) and ionization potential (IP) given in kJ/mol calculated for pelargonidin-3-*O*-glucoside monoanion at M062X/6-31+g(d,p) level in the gas phase, and using SMD solvation models of water and pentyl ethanoate.

Figure S1. HPTLC profiles of 17 samples of six strawberry cultivars after (a) *Bacillus subtillis* bioassay, (b) acetylcholinesterase and (c) butyrylcholinesterase inhibition assays; respective positive controls of each assay are evident at the upper plate part

Figure S2. HPTLC densitogram of the absorbance measurement at 500 nm of 17 samples of six strawberry cultivars

Figure S3. PCs score plots based on chromatogram at white light illumination (a), chromatogram at 254 nm absorbance (b), DPPH• scavenging autogram (c), and *Aliivibrio fischeri* bioautogram (d)

Table S1. Bond dissociation enthalpy (BDE) and ionization potential (IP) given in kJ/mol calculated for pelargonidin-3-*O*-glucoside monoanion at M062X/6-31+g(d,p) level in the gas phase, and using SMD solvation models of water and pentyl ethanoate.

Protonation state	Site	Gas phase		Water		Pentyl ethanoate	
		BDE	IP	BDE	IP	BDE	IP
5–*O* anion	4′-OH	371	674	382	456	371	505
	7-OH	357		383		363	

a) *Bacillus subtillis* bioassay

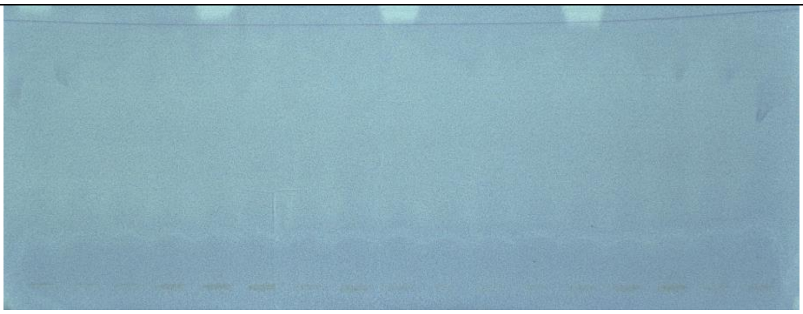

b) Butyrylcholinesterase inhibition assay

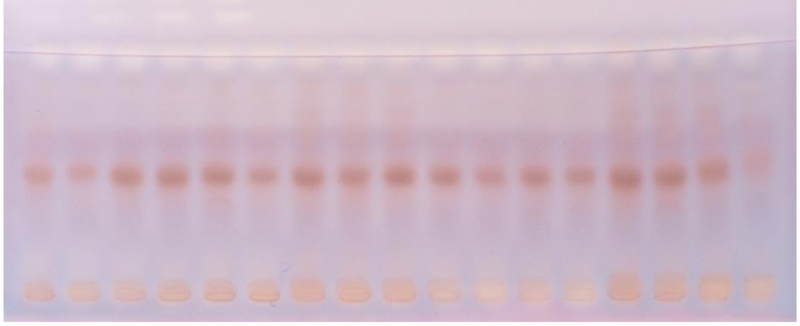

c) Acetylcholinesterase inhibition assay

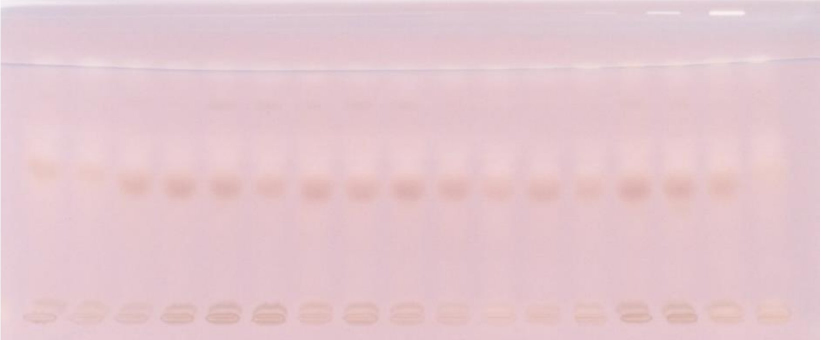

Figure S1. HPTLC profiles of 17 samples of six strawberry cultivars after (a) *Bacillus subtillis* bioassay, (b) acetylcholinesterase and (c) butyrylcholinesterase inhibition assays; respective positive controls of each assay are evident at the upper plate part

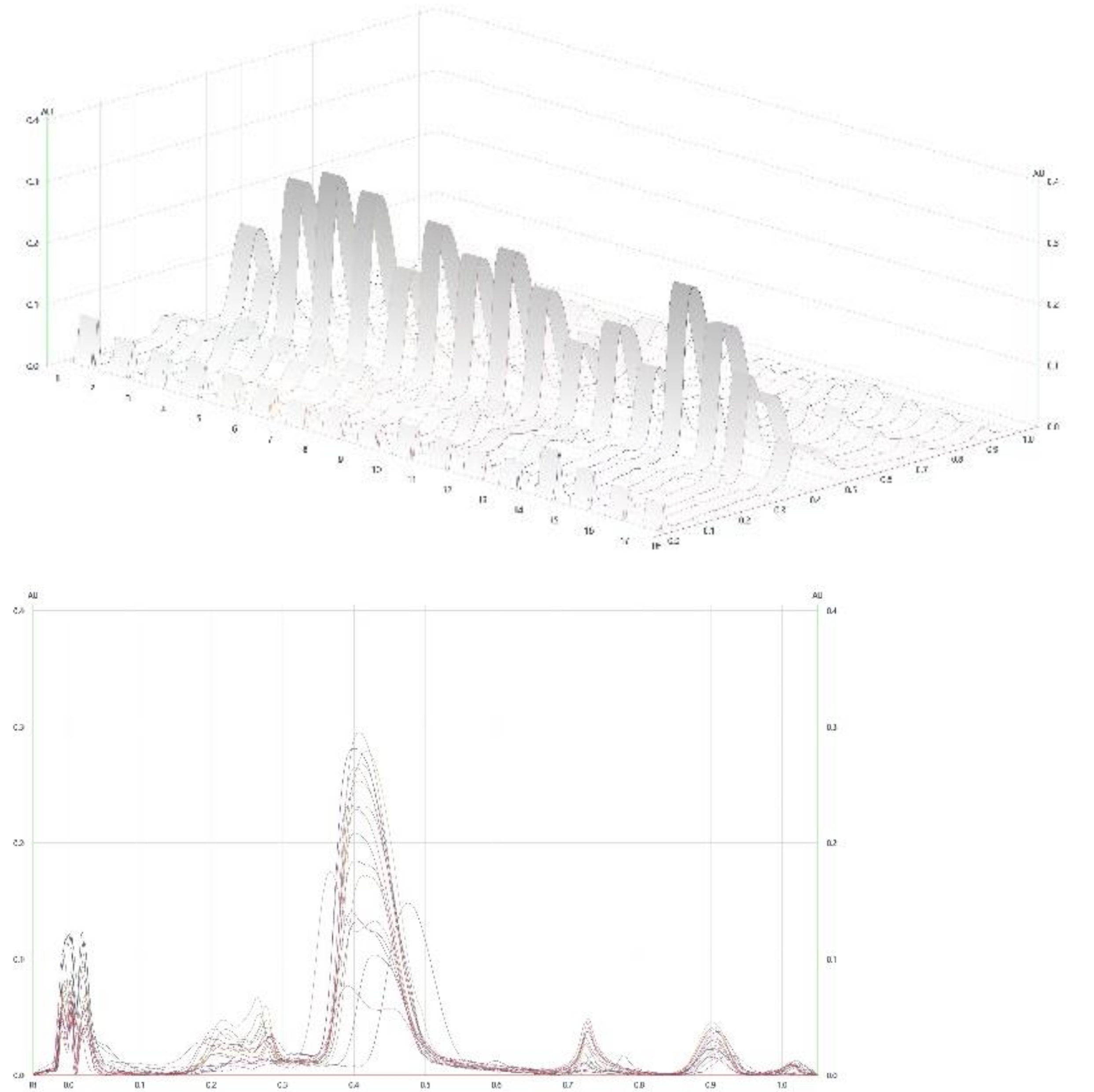

Figure S2. HPTLC densitogram of the absorbance measurement at 500 nm of 17 samples of six strawberry cultivars

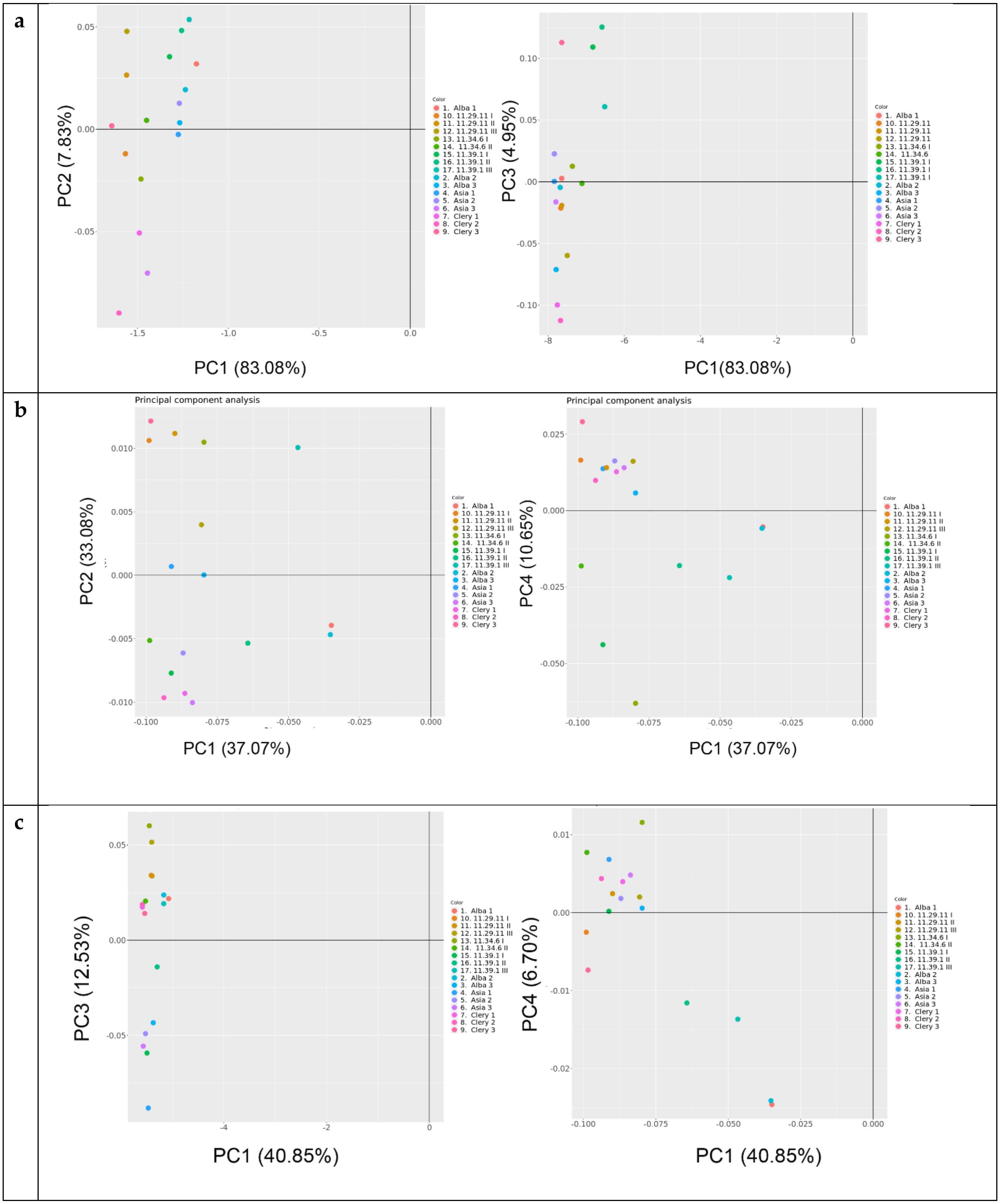

a
PC2 (7.83%)
PC1 (83.08%)
Color
1. Alba 1
10. 11.29.11 I
11. 11.29.11 II
12. 11.29.11 III
13. 11.34.6 I
14. 11.34.6 II
15. 11.39.1 I
16. 11.39.1 II
17. 11.39.1 III
2. Alba 2
3. Alba 3
4. Asia 1
5. Asia 2
6. Asia 3
7. Clery 1
8. Clery 2
9. Clery 3
PC3 (4.95%)
PC1(83.08%)
Color
1. Alba 1
10. 11.29.11
11. 11.29.11
12. 11.29.11
13. 11.34.6 I
14. 11.34.6
15. 11.39.1 I
16. 11.39.1 I
17. 11.39.1 I
2. Alba 2
3. Alba 3
4. Asia 1
5. Asia 2
6. Asia 3
7. Clery 1
8. Clery 2
9. Clery 3
b
Principal component analysis
PC2 (33.08%)
PC1 (37.07%)
Principal component analysis
PC4 (10.65%)
PC1 (37.07%)
Color
1. Alba 1
10. 11.29.11 I
11. 11.29.11 II
12. 11.29.11 III
13. 11.34.6 I
14. 11.34.6 II
15. 11.39.1 I
16. 11.39.1 II
17. 11.39.1 III
2. Alba 2
3. Alba 3
4. Asia 1
5. Asia 2
6. Asia 3
7. Clery 1
8. Clery 2
9. Clery 3
c
PC3 (12.53%)
PC1 (40.85%)
PC4 (6.70%)
PC1 (40.85%)
Color
1. Alba 1
10. 11.29.11 I
11. 11.29.11 II
12. 11.29.11 III
13. 11.34.6 I
14. 11.34.6 II
15. 11.39.1 I
16. 11.39.1 II
17. 11.39.1 III
2. Alba 2
3. Alba 3
4. Asia 1
5. Asia 2
6. Asia 3
7. Clery 1
8. Clery 2
9. Clery 3

d

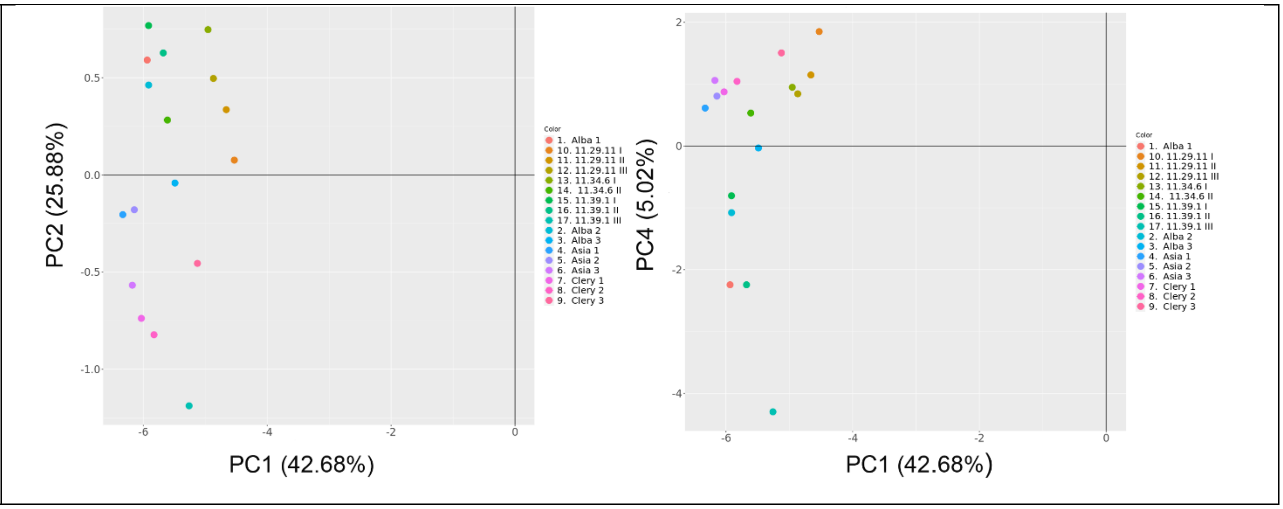

Figure S3. PCs score plots based on chromatogram at white light illumination (a), chromatogram at 254 nm absorbance (b), DPPH• scavenging autogram (c), and *Aliivibrio fischeri* bioautogram (d)
